# Extensive fluvial surfaces at the East Antarctic margin have modulated ice-sheet evolution

**DOI:** 10.1038/s41561-025-01734-z

**Published:** 2025-07-11

**Authors:** Guy J. G. Paxman, Stewart S. R. Jamieson, Neil Ross, Michael J. Bentley, Charlotte M. Carter, Tom A. Jordan, Xiangbin Cui, Shinan Lang, David E. Sugden, Martin J. Siegert

**Affiliations:** 1 Department of Geography, Durham University, Durham, UK; 2 School of Geography, Politics and Sociology, Newcastle University, Newcastle upon Tyne, UK; 3 Alfred-Wegener-Institut, Helmholtz-Zentrum für Polar- und Meeresforschung, Bremerhaven, Germany; 4 British Antarctic Survey, Cambridge, UK; 5 Polar Research Institute of China, Shanghai, China; 6 School of Information and Communications Engineering, Beijing University of Technology, Beijing, China; 7 Institute of Geography, School of Geosciences, University of Edinburgh, Edinburgh, UK; 8 Tremough House, University of Exeter, Penryn, UK

**Keywords:** Geomorphology, Cryospheric science, Palaeoclimate

## Abstract

Antarctic bed topography influences how the overlying ice sheet responds to climate change and provides a record of long-term glacial history. However, knowledge of the processes that governed the development of the landscape before glacial inception and how this modulated subsequent ice-sheet evolution remains limited. Here we use radio-echo sounding to reveal extensive flat surfaces beneath the ice margin between Princess Elizabeth Land and George V Land, East Antarctica. When their elevations are isostatically adjusted for unloading of the present-day ice load, these surfaces cluster at 200–450 metres above sea level and dip gently in an offshore direction. We show that the surfaces are fragments of a once-contiguous coastal plain formed by fluvial erosion, which dates from between the separation of East Antarctica from Australia (~100–80 Ma) and the onset of Southern Hemisphere ice-sheet glaciation (~34 Ma). The preservation of these landforms indicates a lack of intense, selective erosion of the surfaces throughout Antarctica’s glacial history. Fast-flowing ice has instead been directed through inherited tectonic structures and fluvial valleys, leading to the incision of overdeepened subglacial troughs between the flat surfaces and thus modulating the responsiveness of the ice sheet to climate change.

## Abstract (web summary)

Extensive flat surfaces observed beneath the East Antarctic Ice Sheet margin were formed by fluvial erosion and have modulated the ice-sheet response to climate change, according to an analysis of radio-echo sounding data.

## Main

**Fig. 1:**
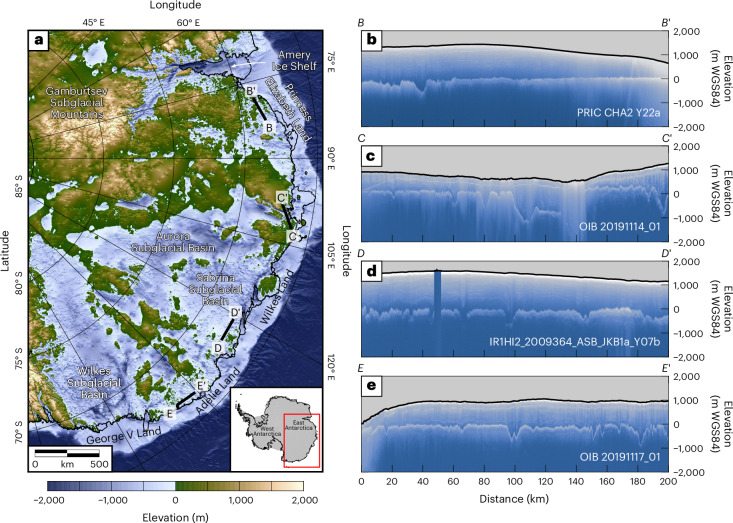
Bed topography of the EAIS. **a**, Bed elevation of the study area (relative to mean sea level) from BedMachine^4^ version 3. Thin black lines mark the modern grounding line and ice calving front. Thick black lines mark the locations of RES profiles shown in panels **b**–**e**. Geographical features mentioned in the text are labelled. Inset shows the study area (red box) within Antarctica. **b**, Elevation-corrected radargram from the 2017 CHINARE (CHA2) survey^24^ (profile *B*–*B*’). **c**, Elevation-corrected radargram from the 2019 Operation IceBridge survey^25^ (profile *C*–*C*’). **d**, Elevation-corrected radargram from the 2009 ICECAP survey^15^ (profile *D*–*D*’). **e**, Elevation-corrected radargram from the 2019 Operation IceBridge survey^25^ (profile *E*–*E*’). In each radargram, the solid line marks the ice surface; the major reflector below is the bed topography. Elevations are relative to the WGS84 ellipsoid. Flight line ID numbers are labelled.

Mass loss from the Antarctic Ice Sheet, and its contribution to global sea-level rise, have accelerated in recent decades^1^. While much focus has been on the West Antarctic Ice Sheet^2,3^, recent studies have highlighted the potential vulnerability of parts of the East Antarctic Ice Sheet (EAIS), which contains ~90% of the 58 metres sea-level equivalent held in Antarctica^4,5^. The EAIS sector between Princess Elizabeth Land and George V Land (70° E to 160° E) is of especial importance because the sub-ice topography contains two large low-lying basins and numerous deep troughs (Fig. 1a)^4^ that may render the ice margin susceptible to rapid and irreversible retreat via marine ice-sheet instability processes^6^. This margin has experienced ice-shelf thinning since the 1970s^7^ and is predicted to be a potentially major contributor to future sea-level rise by numerical ice-sheet models^8–10^. However, substantial uncertainty persists regarding the projected extent and rate of future ice thinning and margin retreat^11^.

Two primary sources of this uncertainty are (1) insufficient knowledge of basal conditions (for example, morphology, temperature, friction and hydrology), which influence ice dynamics and therefore sea-level rise^12^, and (2) limited understanding of long-term EAIS evolution, including its response to past episodes of ocean–atmosphere warming that serve as analogues for contemporary climate change^13^. Addressing both is crucial for improving the ability of numerical ice-sheet models to robustly project future change^14^.

The land surface beneath the EAIS provides a valuable record of ice-sheet behaviour since its inception (~34 million years ago (Ma)) and the geological processes that have shaped the basal environment^15–17^. Across the continents that were formerly connected to East Antarctica in the supercontinent of Gondwana (Africa, India and Australia), the geomorphological record provides the foundation for understanding the tectonic, erosional and climatic history that has influenced long-term landscape development^18^. In Antarctica, airborne radio-echo sounding (RES) surveys are used to measure ice thickness and bed elevation^19^ and generate kilometre-resolution gridded bed products^4,20^ (Fig. 1a). However, the gridding process causes critical geomorphological information to be lost or obscured^21^. Direct measurements of East Antarctic bed topography derived from RES therefore provide a vital opportunity to better understand past, present and future EAIS behaviour^22^.

In this study, we examine RES data from multiple surveys and discover coherent pre-glacial surfaces preserved around the 3,500-km-long margin between Princess Elizabeth Land and George V Land. Analysis of the distribution, elevation and morphology of the surfaces enables us to constrain the basal conditions and long-term behaviour of the EAIS margin and demonstrates that geological processes that operated millions of years ago continue to play a critical role in contemporary EAIS behaviour.

## Distribution and morphology of flat surfaces

**Extended Data Fig. 1:**
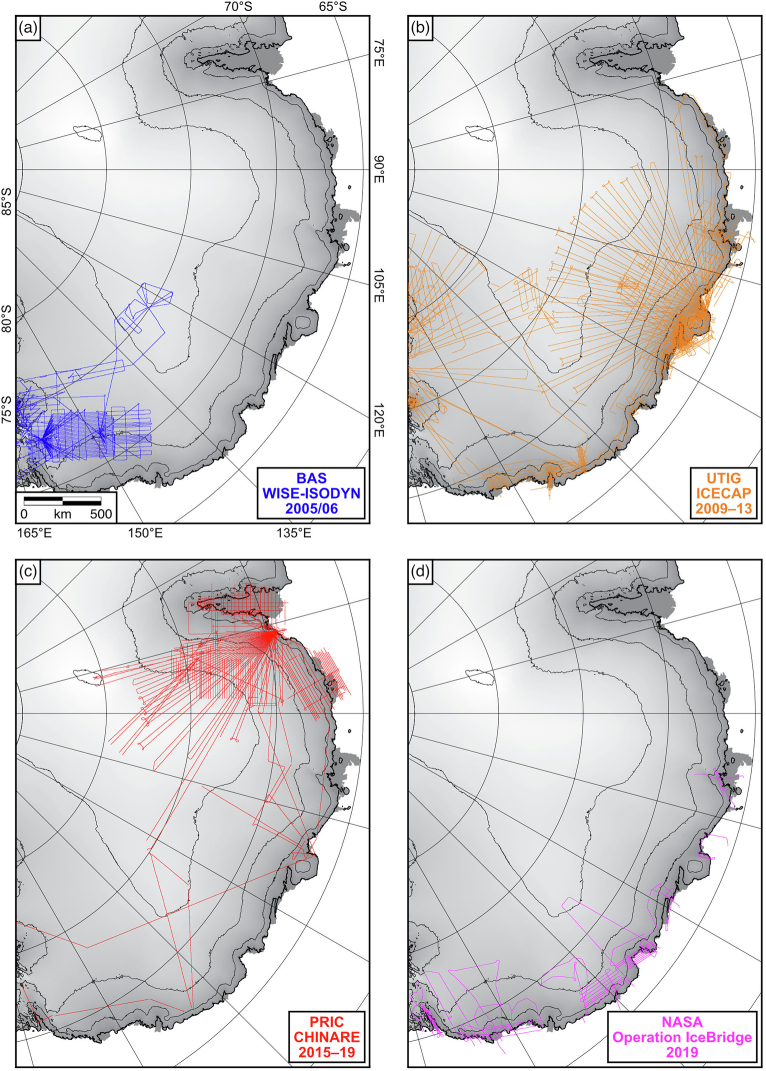
Radio-echo sounding data coverage across the study area. Lines show survey extents for four campaigns: (**a**) WISE-ISODYN (2005–06)^23^, (**b**) ICECAP (2009–13)^15^, (**c**) CHINARE CHA1–4 (2015–19)^24^; survey lines after 2019 are not included, (**d**) Operation IceBridge (2019)^25^; survey lines from pre-2019 were predominantly flown over the Antarctic Peninsula, West Antarctica, and Weddell Sea region, so are not shown here.

To characterize the subglacial topography of the study area, we combined bed elevation measurements from four airborne RES surveys: (1) WISE-ISODYN (Wilkes Basin/Transantarctic Mountains System Exploration–Icehouse Earth: Stability or Dynamism?) 2005–06^23^, (2) ICECAP (International Collaborative Exploration of the Cryosphere through Airborne Profiling) 2009–13^15^, (3) CHINARE (Chinese National Antarctic Research Expedition) CHA1–4 2015–19^24^ and (4) OIB (Operation IceBridge) 2019^25^ (Extended Data Fig. 1). The exact along-track sampling rate depends on the radar platform but is typically ~20 m; the vertical resolution is ~5–10 m (Extended Data Table 1). The coverage of these surveys enabled us to conduct a systematic analysis of the regional bed topography (Methods), which revealed a series of conspicuous low-angle (gradient < 1°), low-relief (internal relief < 200 m) surfaces, hereafter referred to as ‘flat surfaces’.

**Fig. 2:**
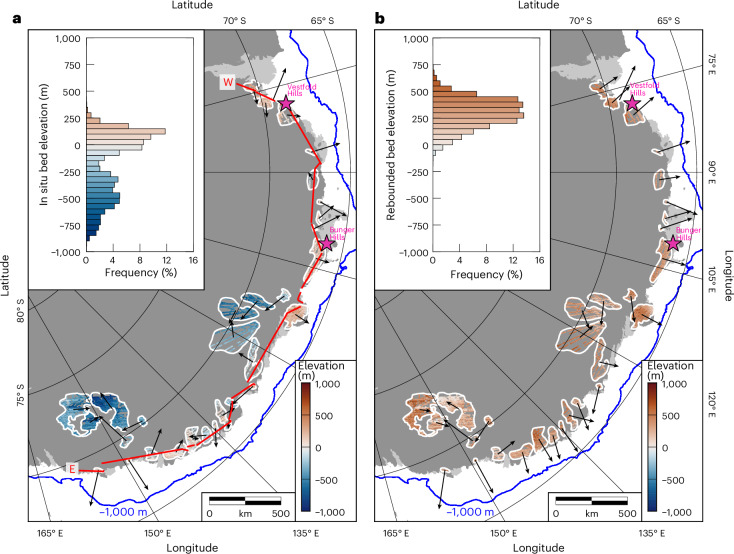
Flat-surface elevations and slopes. **a**, In situ bed elevations of the 31 flat surfaces mapped around the EAIS margin (limits marked in white). Bed elevations were extracted from RES profiles and filtered to remove points that deviate from the local mode by at least ±50 m (that is, valleys or inselbergs). **b**, Rebounded bed elevations of the flat surfaces. Rebounded elevations were computed by adding the in situ elevations to the total isostatic response to the complete removal of the EAIS^27^. All elevations are relative to present-day global mean sea level (the EIGEN-6C4 geoid). Vectors indicate the dip direction of the surfaces, with vector lengths proportional to the dip angle. Blue line marks the −1,000 m bathymetric contour^4^, indicating the position of the continental shelf edge. Insets show the corresponding frequency distributions of elevation (that is, hypsometries). Purple stars mark the surface outcrops in the Bunger Hills and Vestfold Hills. Red line in panel **a** shows 16 RES profile segments used to construct a near-continuous profile around the EAIS margin.

**Extended Data Fig. 2:**
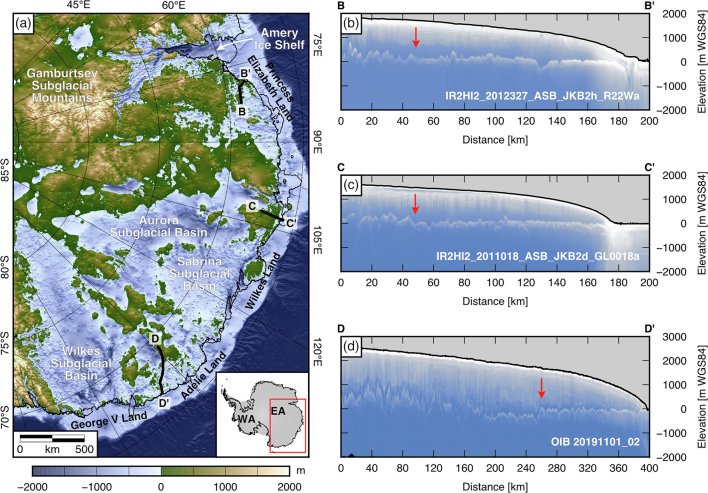
Coast-orthogonal radar profiles crossing the flat surfaces. (**a**) Bed elevation of the study area (as shown in Fig. 1a). (**b**) Elevation-corrected radargram from the 2012 ICECAP survey^15^ (profile B-B’). (**c**) Elevation-corrected radargram from the 2011 ICECAP survey^15^ (profile C-C’). (**d**) Elevation-corrected radargram from the 2019 Operation IceBridge survey^25^ (profile D-D’). The profiles show the transition from the low-relief flat surfaces to the higher, rougher topography on their inland side (inland edges of the flat surfaces are marked by the red arrows). In each radargram, the solid line marks the ice surface; the major reflector below is the bed topography. Elevations are relative to the WGS84 ellipsoid. Flight line ID numbers are labelled.

We mapped 31 flat surfaces around the East Antarctic margin from Princess Elizabeth Land to George V Land (Fig. 2 and Extended Data Table 2). Individual surfaces are characterized by internally consistent elevations and morphologies across 10s to 100s of kilometres, with low-amplitude (<200 m) surficial roughness (Fig. 1b–e). In places, the surfaces are incised by valleys up to 1 km deep and bordered by steep-sided subglacial troughs (Fig. 1). The surfaces range from 200 to 50,000 km^2^ in area and in total comprise ~40% of the perimeter of the EAIS between 70° E and 160° E. On the inland side of the flat surfaces, there is a transition to rougher, higher-elevation topography (Extended Data Fig. 2). The majority of the surfaces are overlain by 500–1,500 m of ice, with a minority located farther into the continental interior and situated beneath ice up to 2,500 m thick^4^ (Supplementary Text 1).

**Extended Data Fig. 3:**
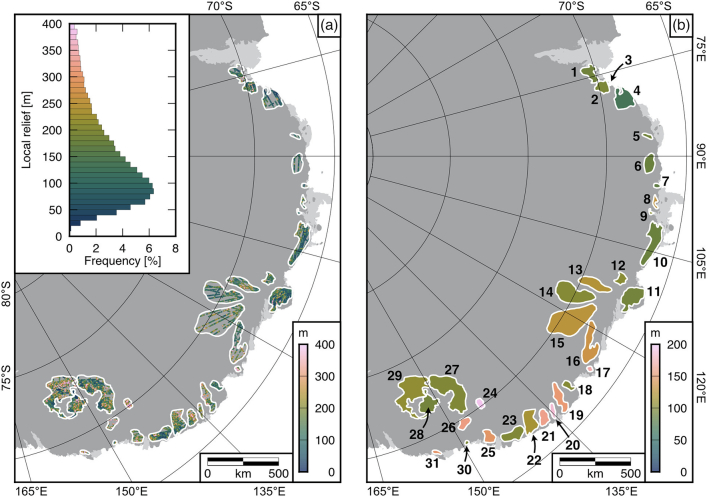
Internal relief within the flat surfaces. (**a**) Local-scale relief of the 31 flat surfaces mapped around the EAIS margin (limits marked in white). Local-scale relief was determined by computing the difference between the moving minimum and moving maximum bed elevations within a 5 km window passed along RES profile segments. Inset shows the corresponding frequency distribution of relief. (**b**) Median local-scale relief for each flat surface. Bold numbers indicate the surface ID numbers (see Extended Data Table 2). Note that the colour scales in the two panels are different.

**Extended Data Fig. 4:**
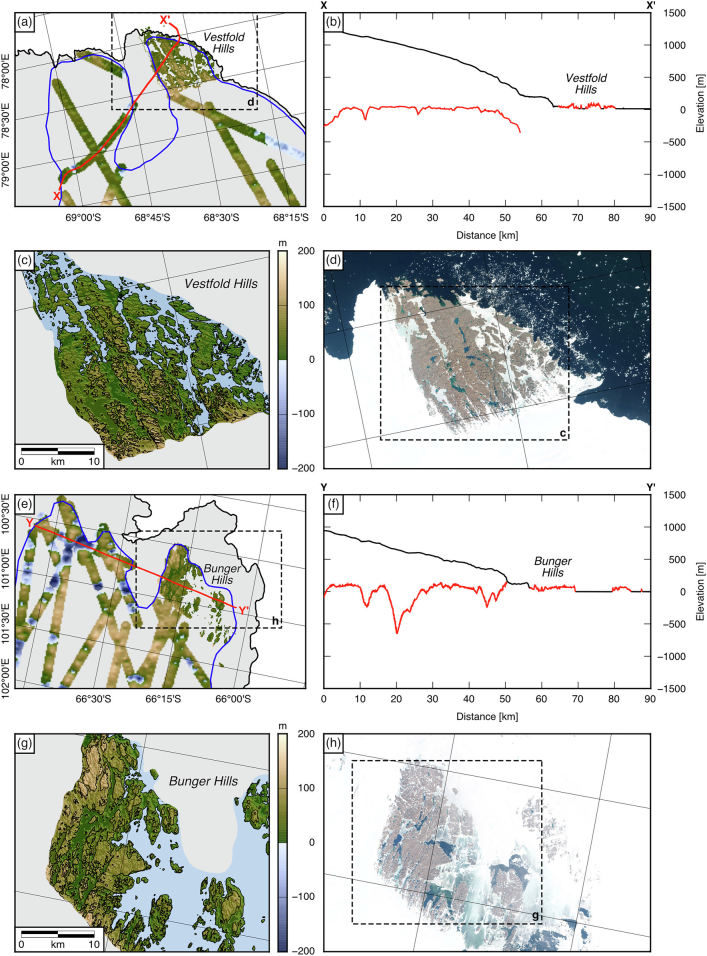
Exposed coastal fragments of flat surfaces in East Antarctica. (**a**) Bed elevation of the exposed Vestfold Hills and contiguous subglacial flat surface. (**b**) Profile of ice surface (black) and bed (red) elevation across the Vestfold Hills along profile X–X’ (location shown in panel **a**). Bed elevation was derived from ICECAP RES survey line IR2HI2_2012327_ICP5_JKB2h_F07T02a; ice surface and land elevation were derived from the Reference Elevation Model of Antarctica (REMA)^26^. (**c**) Hillshaded REMA topography of the Vestfold Hills. Contour interval is 50 metres. (**d**) Sentinel-2b Level-1C imagery of the Vestfold Hills (image extent marked in panel **a**) acquired on 30th January 2024. (**e**) Bed elevation of the exposed Bunger Hills and contiguous subglacial flat surface. (**f**) Profile of ice surface (black) and bed (red) elevation across the Bunger Hills along profile Y–Y’ (location shown in panel **e**). Bed elevation was derived from ICECAP RES survey line IR2HI2_2011002_ASB_JKB2d_GL0152a; ice surface and land elevation were derived from REMA. (**g**) Hillshaded REMA topography of the Bunger Hills. Contour interval is 50 metres (panel extent marked in panel **h**). (**h**) Sentinel-2b Level-1C imagery of the Bunger Hills (image extent marked in panel **e**) acquired on 9th March 2023.

We quantified flat-surface morphology using the RES-derived bed elevation measurements (Fig. 2). Hypsometry (frequency distribution of elevations) shows that, beneath the modern EAIS, the surfaces are situated at a wide range of elevations between −900 and +300 m relative to global mean sea level (Fig. 2a), with a standard deviation of 300 m and a negative excess kurtosis (−0.98). The surfaces are horizontal or gently sloping (<1°) with no systematic alignment in dip direction (Fig. 2a). Across all 31 surfaces, the modal local topographic relief (range of elevations within a 5-km moving window) is ~100 m (Extended Data Fig. 3), with an interquartile range of 80–210 m. Although ice-free land is rare in East Antarctica, isolated coastal outcrops in the Bunger and Vestfold Hills (Fig. 2) exhibit near-identical elevations^26^ and local relief to the adjacent subglacial flat surfaces, and likely represent exposed fragments of the surfaces on their coastward edges (Extended Data Fig. 4).

**Extended Data Fig. 5:**
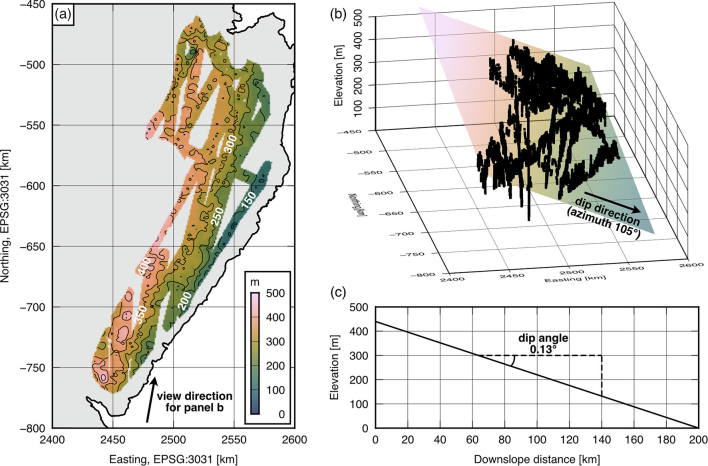
Determination of the dip direction and angle of flat surfaces. (**a**) Modal filtered, ice-free (rebounded) bed elevation of a flat surface. Contour interval is 50 m. (**b**) Fitting of a 2D plane to all elevation points within the flat surface (black) via linear regression. Colour scale matches that shown in panel a. (**c**) The angle and azimuth of steepest descent of this plane were extracted and used to compute the surface-averaged dip angle and dip direction.

Notably, when flat-surface elevations are isostatically adjusted for the complete removal of the EAIS^27^ (Methods and Supplementary Text 1 and 2), the hypsometric distribution is characterized by a well-defined modal peak at +200–450 m (Fig. 2b) and a reduced standard deviation of 135 m. The hypsometric distribution has a positive excess kurtosis (0.57) and a skewness of −0.21; this asymmetry reflects the presence of valleys incised into the flat surfaces (Fig. 1). Moreover, when adjusted for EAIS removal, the dips of the surfaces systematically align orthogonal to the coast, with gentle coastward dip angles of up to 0.8° (Fig. 2b and Extended Data Fig. 5). This applies to all surfaces proximal to the coast; the surfaces farther into the interior of the Wilkes and Sabrina Subglacial Basins show a more complex pattern of dips, which may reflect differences in inherited morphology, erosive history and/or local tectonic deformation.

**Fig. 3:**
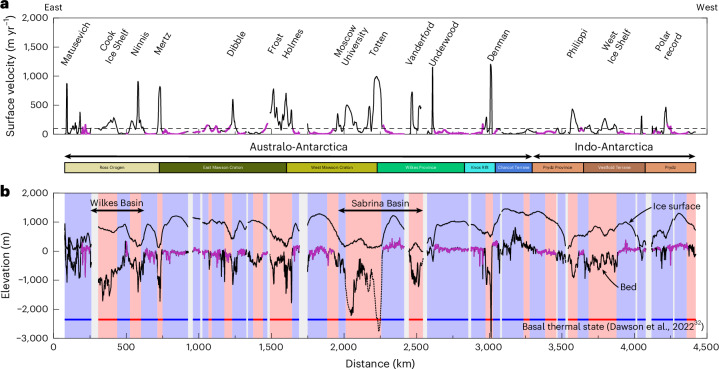
Transect of the EAIS margin. **a**, Ice-surface velocity^30^ along the profile displayed in Fig. 2a, with major outlet glaciers labelled. Horizontal dashed line marks the 100 m yr^−1^ contour, an approximate threshold of fast flow. **b**, Ice surface and bed elevation from 16 RES profiles used to construct the transect. Flat surfaces are highlighted in purple. Dashed lines mark sections of bed overlain by floating ice shelves; sub-shelf bathymetry from BedMachine^4^ version 3. Red/blue shading indicates the model-predicted basal thermal state of the EAIS^32^; red, basal ice within 2 °C of the pressure melting point (that is, ‘warm-based’); blue, basal ice >2 °C below the pressure melting point (that is, ‘cold-based’). Horizontal bars between panels **a** and **b** mark the extents of the major geological provinces along the transect^28,29^.

**Extended Data Fig. 6:**
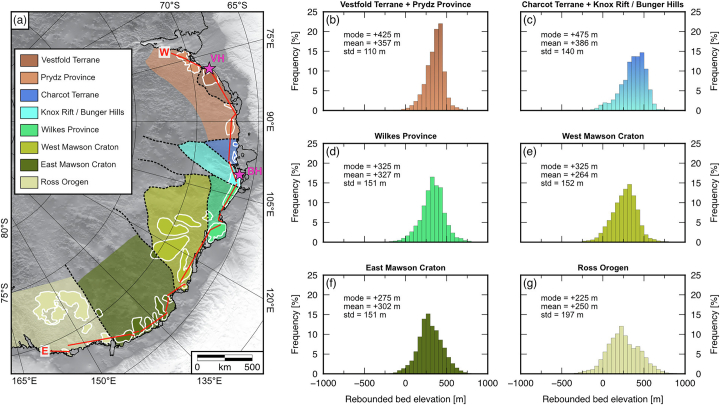
Geological provinces around the East Antarctic margin. (**a**) Map of first-order geological provinces^28,29^. Dashed lines mark boundaries between the provinces. Purple stars mark the flat surface outcrops in the Bunger Hills (BH) and Vestfold Hills (VH). Red line shows 16 RES profile segments used to construct a near-continuous profile around the EAIS margin (Fig. 3). (**b–g**) histograms of the rebounded bed elevations of the flat surfaces within each of the geological provinces. For simplicity, the Vestfold Terrane and Prydz Province have been grouped together (**b**) as have the Charcot Terrane and Knox Rift / Bunger Hills (**c**). The modal and mean flat surface elevations for each province are labelled, as is the standard deviation.

**Extended Data Fig. 7:**
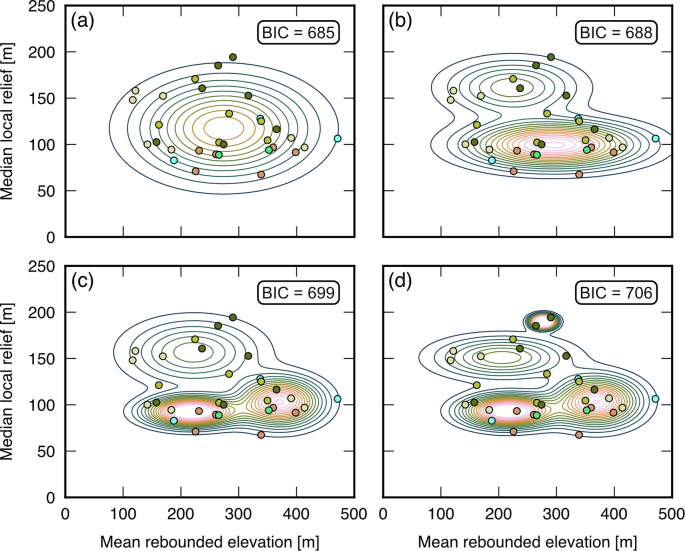
Gaussian Mixture Model cluster analysis of the flat surfaces. The morphology of each flat surface is quantified by its mean rebounded (ice-free) elevation and median local relief. (**a**) One-component (*k* = 1) mixture model, (**b**) Two-component (*k* = 2) mixture model, (**c**) Three-component (*k* = 3) mixture model, (**d**) Four-component (*k* = 4) mixture model. Circles represent the 31 flat surface fragments mapped in this study (colours correspond to geological provinces; see Extended Data Fig. 6). Contours represent the probability distribution of the Gaussian component(s) in each of the four mixture models. BIC = Bayes Information Criterion; a lower BIC indicates a better-fitting model (see Methods).

Bedrock geology does not appear to exert a strong control on flat-surface elevation or morphology in East Antarctica. Regional geology comprises primarily Archaean–Proterozoic crystalline gneisses, with intervening Palaeozoic–Mesozoic orogenic belts, igneous intrusive provinces and rift basins^28,29^. Flat surfaces are present within each geological province, and some surfaces cut across geological boundaries (Fig. 3 and Extended Data Fig. 6). While we note a slight increase in modal flat-surface elevation moving westwards around the margin (Extended Data Fig. 6), Gaussian mixture model cluster analysis indicates that the flat surfaces comprise a single, statistically consistent, population according to their elevations and morphologies (Methods and Extended Data Fig. 7).

**Extended Data Fig. 8:**
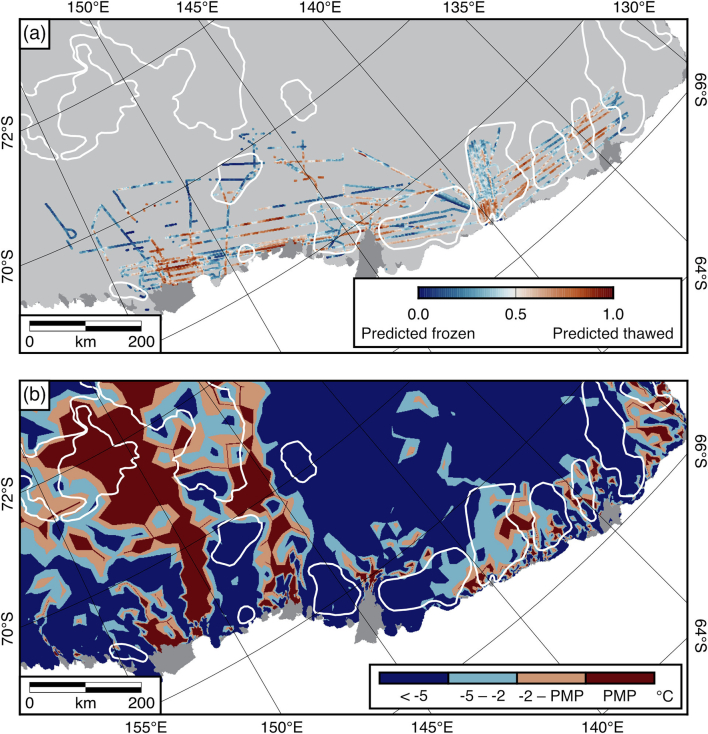
Basal thermal state of the EAIS in George V Land and Adélie Land. (**a**) Predicted basal thermal state along RES lines^31^. Colours represent the probability of the basal ice being frozen (0) or thawed (1); the map shown is an aggregate of four different combinations of labelling and radar processing^31^. White polygons mark the flat surfaces. (**b**) Predicted basal temperatures derived from simulations of the present-day Antarctic Ice Sheet generated using the Ice-sheet and Sea-level System Model (ISSM)^32^. Temperatures are divided into four bins: (i) at the pressure melting point (PMP), (ii) less than 2 °C below the PMP, (iii) between 2° and 5 °C below the PMP, and (iv) more than 5 °C below the PMP.

Ice-surface velocities above the flat surfaces are consistently low; only five surfaces have a mean overlying flow speed of >50 m yr^−1^, and none exceeds 100 m yr^−1^ (ref. ^30^). Fast ice flows around the flat surfaces and through the intervening deep troughs that host major outlets, including the Mertz, Totten and Denman Glaciers (Fig. 3a). Observational constraints from radar attenuation rates and bed-echo power in George V Land and Adélie Land indicate that the flat surfaces in these regions are typically situated beneath ice that is probably cold-based (that is, frozen bed) whereas the adjacent troughs probably host warm-based ice (that is, thawed bed;^31^ Extended Data Fig. 8). Ice-sheet model predictions of modern basal temperatures also indicate that the basal thermal state of the ice overlying the flat surfaces is cold-based, with warm-based ice in the intervening topographic lows^32^ (Fig. 3b).

## Flat-surface formation

We interpret the flat surfaces to have originated as pre-glacial fluvial planation surfaces. The reasons for this are threefold. First, the strong clustering of rebounded elevations around the entire margin (Fig. 2b) is indicative of surface formation via a single process operating in the absence of the ice sheet. Second, the gentle coastward dips of the surfaces (Fig. 2b) are consistent with coastal plains formed by planation to base level by river systems, as observed along former Gondwanan passive margins such as southern Australia and southwest Africa^33^. Third, the flat surfaces cut across the regional geological structure (Fig. 3b), indicating that they are not the expression of an exhumed geological surface such as an erosion-resistant stratigraphic horizon.

We discount both marine and ice-sheet planation as primary formation mechanisms for the flat surfaces as there are no modern analogues for the generation of coherent, morphologically consistent flat surfaces over such large distances (3,500 km) in an inland setting via these processes. The surfaces extend too far inland and above sea level to have been eroded by wave action or truncated by grounding-zone/ice-shelf fluctuations, and neither process can account for the consistent coastward overall dips of the surfaces. We also discount the surfaces being sediment-draped depositional features because of their observed high-frequency, low-amplitude roughness (Fig. 1b–e). This is indicative of erosion of coherent bedrock. Indeed, the exposed continuations of the flat surfaces in the Bunger and Vestfold Hills comprise Archaean–Proterozoic crystalline gneisses^34^.

**Fig. 4:**
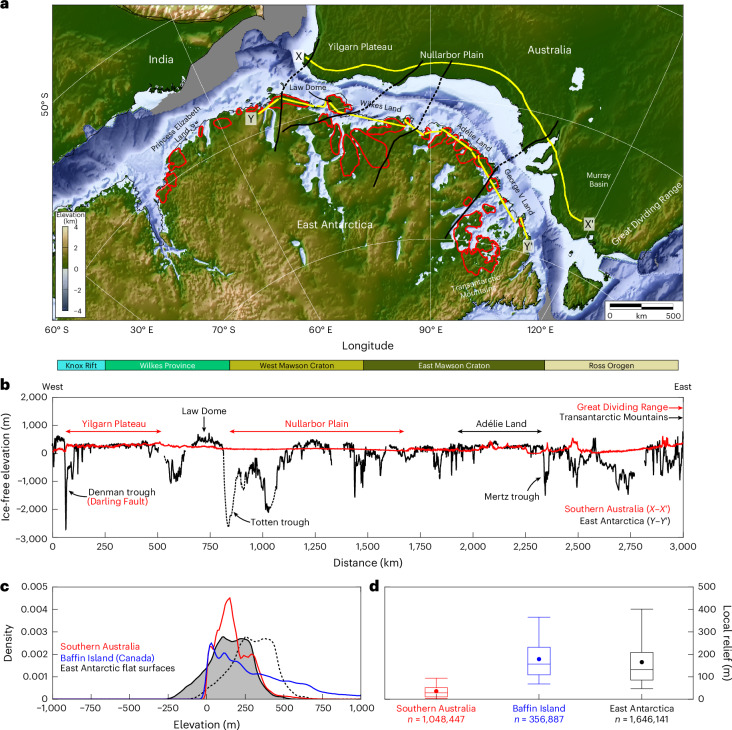
Comparison of the southern Australian and East Antarctic conjugate margins. **a**, Palaeogeographic reconstruction of India, Australia and East Antarctica at 157 Ma (ref. ^58^). Labels indicate modern-day topographic features. Red polygons mark the flat surfaces around the East Antarctic margin. Black lines mark major geological boundaries correlated between Australia and East Antarctica using potential field data^29^. **b**, Transects of southern Australian margin topography (red) and East Antarctic margin bed topography (black; adjusted for ice-sheet unloading). Profile locations are indicated by the yellow lines in panel **a**. Horizontal bars between the panels mark the extents of the major geological provinces along the transect^29^, delineated by the black lines shown in panel **a**. The Indian continental margin is not analysed due to the topographic effects of the Himalayan Orogen. **c**, Kernel density estimation plot of the rebounded topography of the East Antarctic flat surfaces. Dashed line represents rebounded topography (compare Fig. 2b); solid line with shading represents rebounded topography shifted by −150 m. Red and blue lines show equivalent plots for the topography of southern Australia (fluvial passive margin) and Baffin Island (glaciated passive margin), respectively. **d**, Box plots of local-scale relief for the three regions shown in panel **c**, calculated using *n* individual point measurements. Box plots mark the mean (circle), median (central line), interquartile range (box) and 5th and 95th percentiles (outer bars).

The first-order topography of this sector of the East Antarctic margin closely resembles that of its conjugate in southern Australia. Both are characterized by low elevations and gradients along much of their length and high terrain at their eastern end (Fig. 4). The narrow, unimodal hypsometric distributions of both margins are also closely comparable (Fig. 4c). As is the case for many passive margins, the southern Australian margin (which has escaped Cenozoic glaciation) is characterized by a low-relief coastal plain, which formed via long-term fluvial planation^18^ following its separation from East Antarctica. We suggest a similar fluvial origin for the flat surfaces mapped along the East Antarctic conjugate margin.

Although we interpret the first-order morphology (that is, elevations and overall dips) of the flat surfaces as being fluvial in origin, we emphasize that the second-order morphology (that is, surficial roughness) was probably generated by ice. This is evidenced by the geomorphology of the Bunger and Vestfold Hills, which closely resemble ‘knock-and-lochan’ topography formed via the removal of regolith and ‘etching’ of the bedrock surface by areal scouring beneath warm-based ice^35,36^ (Extended Data Fig. 4). The local relief of the subglacial surfaces (80–210 m) is ~100 m larger than the local relief of the southern Australian conjugate margin (Fig. 4d) and is consistent with the exposed outcrops in the Bunger and Vestfold Hills. In landscapes of areal scour elsewhere on Earth (for example, former ice-sheet beds such as Baffin Island; Fig. 4d), the depth of scouring is <100 m (ref. ^36^). This indicates that the flat surfaces have been subjected to scouring, which increased their internal relief by up to 100 m.

Scouring of the flat surfaces by warm-based ice probably occurred earlier in the history of the EAIS. This is evidenced by the presence of localized Pliocene (~4 Ma) shallow marine deposits that drape the coastal surfaces in the Vestfold (and neighbouring Larsemann) Hills and are not scoured^37^, which shows that the scouring (and the flat surfaces themselves) pre-date the Pliocene. Although it is difficult to further constrain the age of scouring, there is evidence for limited EAIS erosion under a progressively arid polar climate since the mid-Miocene^5,22^. We suggest that the erosive events occurred under the more dynamic ice sheets of the Oligocene/early Miocene^38,39^, although more recent erosive modification cannot be discounted.

Selective incision of deep glacial troughs divided the once-contiguous surface into the multiple coherent fragments observed today (Fig. 3b). This selectivity of glacial erosion is evidenced by the correlation between the positions of these troughs and the thickest sequences of post-34 Ma offshore sediment^40^. Indeed, the continental shelf width (a proxy for the amount of sediment progradation) is narrower in front of the flat surfaces than the intervening deep troughs (Fig. 2) (ref. ^41^), indicating that the surfaces have not experienced substantial glacial erosion. The continuity of the flat surfaces either side of the intervening deep troughs (Fig. 3b) also indicates that they pre-date trough incision. This incision probably commenced soon after ~34 Ma (ref. ^42^), and possibly several million years earlier^38^, which places a lower bound on the timing of original surface planation by fluvial systems.

**Fig. 5:**
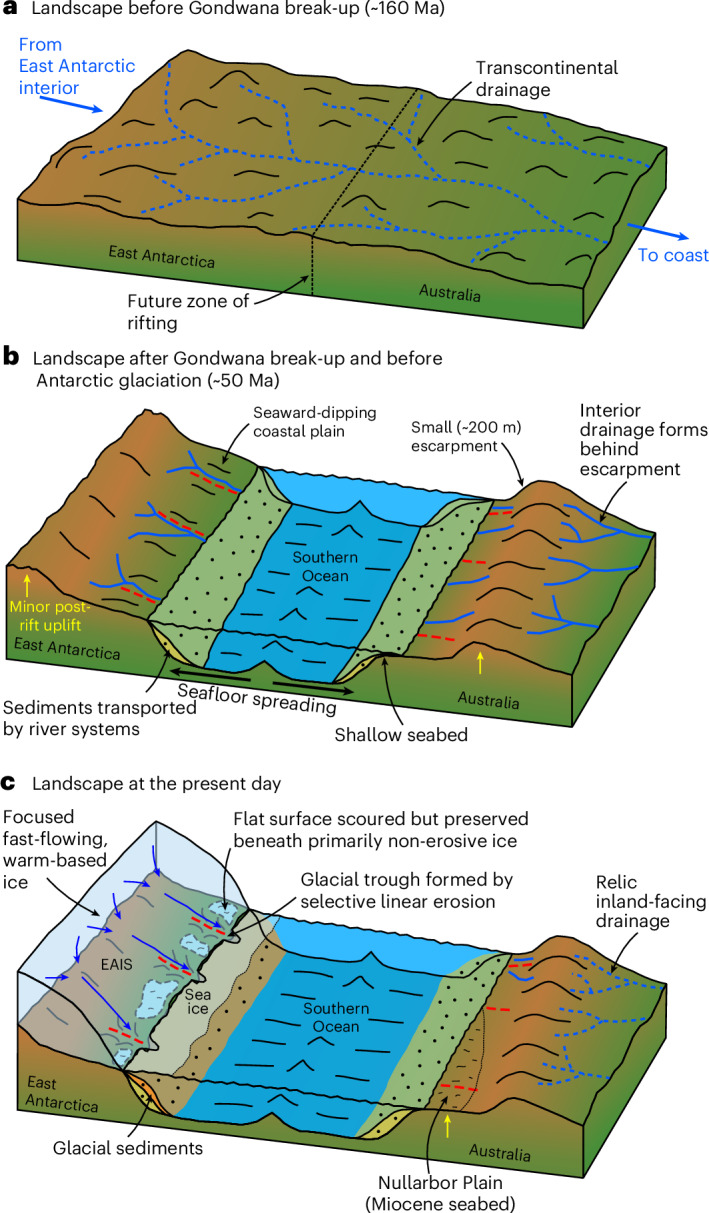
Schematic of Australo-Antarctic margin evolution. **a**, Before Gondwana break-up (pre-160 Ma), transcontinental fluvial drainage systems flowed from the highlands of interior East Antarctica to the northwestern coast of Australia^43^. **b**, Following continental break-up and seafloor spreading onset at ~80 Ma, a small (~200 m) escarpment formed along parts of the southern Australian margin, and an interior drainage network was established on the inland side. In East Antarctica, rivers eroded down to the new base level, forming a low-lying, seaward-dipping coastal plain. **c**, After ~34 Ma, Australia’s climate became increasingly arid, and the interior drainage network became extinct. A limestone seabed became subaerially exposed during the Miocene, forming the Nullarbor Plain^59^. Following ice-sheet growth, remnants of the East Antarctic fluvial planation surface were surficially scoured by ice, but the first-order inherited fluvial structure was preserved due to prevailing low subglacial erosion rates in these areas. Contemporaneously, fast-flowing, warm-based ice selectively exploited existing structural weaknesses (red dashed lines) and/or river valleys to erode deep glacial troughs between the flat-surface fragments.

Sediment provenance studies show that, before break-up, trans-Gondwanan fluvial pathways extended from central East Antarctica to northwestern Australia (via Wilkes Land and southwestern Australia; Fig. 5a)^43^. This indicates that there was early connectivity of the already relatively low-lying Antarctic and Australian fluvial landscapes, which may have started to develop as early as the Triassic period^44^. Relative motion between the continents commenced at ~160 Ma, and seafloor spreading onset is dated at ~100–80 Ma (ref. ^45^). Following continental break-up, minor post-rift uplift and the formation of a new (lower) base level would have triggered enhanced erosion, with fluvial systems grading the landscape to base level as a seaway formed between East Antarctica and Australia. We envisage that this process formed a contiguous coastward-dipping planation surface around the East Antarctic margin, as well as reorganization of pre-break-up fluvial pathways. Indeed, the Yilgarn Plateau in southwestern Australia (Fig. 4a) contains palaeochannels of middle Eocene age (~45 Ma) with an inland-facing palaeo-drainage direction^46^, a pattern probably caused by minor escarpment uplift (Fig. 5b). We therefore infer that East Antarctic flat-surface planation post-dated Australia–Antarctica separation (~80 Ma) and pre-dated EAIS inception (~34 Ma).

The seaward edges of the East Antarctic flat surfaces (under ice-free conditions; Fig. 2b) are consistently ~150 m above present-day sea level, providing an approximate indication of base level at the time of formation. Although the surfaces may have subsequently been vertically displaced by processes including post-rift thermal subsidence and the isostatic response to glacial trough erosion and offshore sediment deposition, calculations show that the contributions of these processes would have been minor (<50 m; Supplementary Text 3–5). Moreover, these processes generate displacements with opposite signs that partially cancel out. Importantly, these processes cannot account for the consistent elevations and gentle coastward dips of the flat surfaces. A base level 150 ± 50 m higher than today is in broad agreement with estimates of global sea level for the Palaeocene to early Eocene (~66–48 Ma; Supplementary Text 2)^47,48^.

## Implications for ice-sheet dynamics

Our findings demonstrate that tectonic and surface processes that operated millions of years ago have been crucial in preconditioning the behaviour of the EAIS margin, including its most sensitive outlets. Contemporary ice flow is focused through the deep troughs between the remnants of the pre-glacial fluvial planation surface^30^ (Fig. 3). Inherited geological structures and/or weaknesses may have been exploited by pre-glacial river valleys and in turn by early outlet glaciers when the EAIS first expanded to continental scale^38^. Preferential steering of ice and basal meltwater^49,50^ would facilitate selective incision of existing topographic lows to form the overdeepened troughs observed today (Fig. 5). The deep troughs now host fast-flowing glaciers such as Totten, Mertz and Denman (Fig. 3), which are vulnerable to retreat via marine ice instability feedbacks owing to their topography^4^.

The presence of extensive pre-glacial surfaces between the deep troughs has important implications for long-term ice behaviour. To have preserved these surfaces with minor modification (that is, scouring) since at least ~34 Ma, the long-term average basal conditions of these parts of the EAIS must have been conducive to low erosion rates (that is, cold-based and/or slow-moving; Fig. 5)^51^. Generation of high-frequency, low-amplitude roughness across the surfaces via areal scour can only have been short-lived and/or intermittent; otherwise, the surfaces would not exhibit such consistent rebounded elevations and coastward dips around the entire margin. The surfaces therefore act as a new constraint for basal thermal state around the EAIS margin, which is one of the key factors that govern the rate of basal sliding and onset of fast ice flow but is poorly understood and differs between ice-sheet models^31^.

The observation that the sub-ice flat surfaces are contiguous with outcrops in the Bunger and Vestfold Hills enables us to further constrain basal conditions across a large, grounding-zone proximal sector of the EAIS margin. On the basis of the geology of these exposed areas^52^, we infer the presence of hard, crystalline bedrock across the flat surfaces adjacent to these outcrops. These lithologies are typically associated with relatively high basal friction, low permeability and low erodibility^12^. Other flat surfaces, particularly those within Wilkes and Sabrina Subglacial Basins, may instead comprise coherent sedimentary rock^29^.

We hypothesize that the flat surfaces would play a stabilizing role during past and future grounding-zone retreat. As the ice margin retreats and thins, increased flotation of ice may occur within the low-lying troughs (Fig. 3). However, ice would probably remain grounded on the flat surfaces, which stand 1–2 km higher than the adjacent troughs (Fig. 3b), resulting in the formation of ice rises^53^. Grounding may be reinforced as the flat surfaces increase in elevation due to the regional isostatic response to ice unloading, depending on the rebound rate^27,53^. Indeed, ice rises underlain by flat-topped bathymetric highs are observed in the Weddell and Ross Sea embayments^21,53,54^, and some flat surfaces mapped in this study currently host ice rises, including the Law Dome (Fig. 4)^53^. Ice rises act as pinning points and buttress the inland ice, reducing mass flux to the ocean and inhibiting grounding-zone retreat^55,56^, and can also increase local accumulation rates^57^. However, ice-rise formation may not be fully captured in continental-scale ice-sheet models^8–10^, particularly if ice shelves are not retained during grounding-zone retreat. Limited model resolution and/or uncertainties surrounding the parameterization of processes that determine grounding-zone position (for example, marine ice-sheet instability, ice-cliff calving, ice-shelf hydrofracturing and glacial isostatic adjustment) may also contribute to this issue^6^. The spatial distribution of the mapped flat surfaces may facilitate the formation of ice rises capable of slowing the rate of grounding-zone retreat during warmer climates.

We emphasize the need for further exploration of the influence of the flat surfaces on ice dynamics during past warmer climates, for example, through acquisition of offshore records, onshore bedrock sampling and testing of numerical ice models that apply appropriate physics at a resolution sufficient to resolve grounding-zone dynamics and ice-rise formation. This will help further resolve the nature of past EAIS margin retreat, with important implications for projections of future ice change and sea-level rise in a warming world.

## Methods

### RES data compilation

We used RES data to map subglacial flat surfaces around the margin of East Antarctica between Princess Elizabeth Land (70° E) and George V Land (160° E). We used geolocated ice thickness data acquired during four airborne RES surveys:

1. WISE-ISODYN^23^
2. ICECAP^15^
3. CHINARE^24^
4. OIB^25^

Given the close similarity of the along-track sampling rates and vertical resolutions of each of the four survey platforms (Extended Data Table 1), all along-track ice thickness measurements were integrated into a single database with no additional processing. The latitude and longitude of each sample point were projected into polar stereographic (EPSG:3031) eastings and northings. These coordinates were in turn used to compute the along-track Cartesian distance for each survey line. Bed elevations were determined by subtracting ice thickness from the ice-surface elevation; for each survey, elevations are referenced to the WGS84 ellipsoid. To ensure all bed elevations were referenced to global mean sea level, we shifted them onto the EIGEN-6C4 geoid using the BedMachine Antarctica geoid height correction^4^. To ensure consistency across the entire dataset and to avoid aliasing of high-frequency variations in elevation, we upsampled the bed elevation data along each survey line to a uniform along-track spacing of 10 m.

### Flat-surface identification and mapping

To locate candidate flat surfaces, we performed a first-pass visual inspection of each survey line in the compiled bed elevation dataset and noted sections of the profiles characterized by coherent, low-relief, low-angle topography. We then fitted a linear polynomial (in the least-squares sense) to each of these one-dimensional (distance versus elevation) profile segments, which ranged in length from ~10 to ~200 km. This polynomial was used to estimate the overall gradient from the start to the end of the profile. We imposed a condition that any segment with a gradient exceeding 1° was excluded from our inventory. We note that the flight lines have a range of azimuths (Extended Data Fig. 1) and will typically not be oriented along the direction of steepest gradient of a two-dimensional topographic surface, but because this will result in an underestimation of the gradient at this stage, ‘borderline’ low-angle surfaces will be included (rather than excluded) in this first-pass compilation.

We also estimated the local-scale relief within the candidate flat surfaces by computing the moving minimum and moving maximum elevations within a 5 km window passed along the profile segments. The difference between the moving minimum and maximum provides an indication of the local-scale (that is, <5 km) relief (that is, range in elevations) across the flat-surface segments. We imposed a condition that any segment with a median local relief exceeding 200 m was excluded. Any segment not excluded by these two conditions was considered to be a segment of a low-angle, low-relief (that is, ‘flat’) surface. In total, we identified 538 such segments within the full RES dataset. We then plotted the spatial extent of these segments and manually digitized polygons demarcating the two-dimensional extent of 31 separate flat surfaces within the study area.

### Flat-surface morphological characterization

To characterize flat-surface morphology, we extracted the bed elevation points from the 538 segments, each of which is assigned to one of the 31 separate flat surfaces. Each segment profile was detrended by subtracting a linear polynomial (fitted in the least-squares sense) from the observed bed elevations. We then identified the modal detrended elevation by grouping the elevations into 10 m bins. The segments were then modal filtered by removing any point deviating from the mode by more than 50 m. These points constitute valleys incised into the modal surface or peaks protruding above it. The remaining points were considered to represent the contiguous flat surface itself and were restored to their true elevations by re-adding the linear polynomial. These restored modal-filtered elevations were used to determine the hypsometry—the frequency distribution of elevation across all the flat surfaces—using a bin size of 50 m.

We then determined the average dip of each of the 31 flat surfaces by fitting a two-dimensional plane to all modal-filtered elevation points within each polygon via linear regression. By including all the elevation point data within each surface and fitting a two-dimensional plane, we addressed the issue of individual flight lines not recording the ‘true dip’ of the surface. Although the flight lines are not isotropic, each flat surface is sampled by multiple flight lines oriented in multiple directions (for example, Extended Data Fig. 5), and the fitted planar surfaces are therefore sufficiently well constrained to robustly extract the true dip. The angle and azimuth of steepest descent of the plane were extracted as the overall dip and dip direction of the surface, respectively. We also computed the along-track local-scale relief and the median value within each of the 31 flat surfaces, using the method described in the previous section.

We then repeated the preceding analysis for bed elevations that were adjusted for the isostatic response to the complete unloading of the Antarctic Ice Sheet. To do so, we used a recent calculation of the isostatic response to complete deglaciation^27^, which was computed using a flexed elastic plate model^60^, BedMachine v.3 ice thickness^4^, and a laterally variable effective elastic thickness of the lithosphere^61^. Although the continent-scale ice thickness grid will deviate from the local radar-derived ice thickness, these differences will be minor (<100 m), and the flexural response to ice unloading is a long-wavelength signal, so the local differences in flexure are negligible. The correction also accounts for the equilibration of the ongoing response to Antarctic ice-mass change since the Last Glacial Maximum and feedbacks associated with loading of areas below sea level by water. For simplicity, rebounded bed elevations are referenced to present-day global mean sea level.

For comparison, we also computed the hypsometry and local-scale (5-km-window) relief for southern Australia (a fluvial passive margin) and Baffin Island, Canada (a glaciated passive margin) using the same methods as described for East Antarctica. In the case of Baffin Island, the value of using a former ice-sheet bed in the Northern Hemisphere is that it represents the effects of glacial modification (via areal scouring) of an existing landscape. Topography data for these regions were taken from the Copernicus global 90-m-resolution digital elevation model^62^. We note that the conjugate margin for the flat surfaces in Princess Elizabeth Land is the eastern side of the Indian subcontinent (Fig. 4), but this area (modern-day Bangladesh) is dominated by large volumes of Cenozoic sedimentation in the Himalayan foreland basin, meaning there is no longer any meaningful comparison with the topography of East Antarctica.

### Cluster analysis

To determine the similarity in morphology between the 31 flat surfaces and to ascertain whether the surfaces could be divided into multiple statistically distinct groups, we performed cluster analysis using the Gaussian mixture model^63^. The Gaussian mixture model is a probabilistic model that assumes that an *N*-dimensional sample of data can be described by a mixture of a finite number, *k*, of Gaussian distributions with unknown parameters. It therefore allows the number of statistically distinct clusters within the dataset to be identified. For this modelling, we assembled a two-dimensional dataset with 31 entries, comprising the mean rebounded elevation and the median local relief for each flat surface.

The Gaussian mixture model implements the expectation-maximization algorithm, an iterative process that assigns each datapoint to a cluster by maximizing the posterior probability that the datapoint belongs to its assigned cluster. We initialized the model by choosing starting means and covariances for the *k* Gaussian components using the k-means++ algorithm^64^. This selects the mean of the first component at random from the data and then chooses subsequent means from a weighted distribution of the data, favouring points farther away from the existing means. Initial mixing proportions are assumed to be uniform. The model first computes the probability of each point having been generated by each of the initialized Gaussian components. The parameters and mixing proportions of the Gaussian components are then adjusted iteratively to maximize the likelihood (posterior probability) across all datapoints. We allowed a maximum of 500 iterations to ensure convergence to a local optimum and restricted the covariance matrices to be diagonal.

To identify the optimum number of components to describe the flat-surface dataset and thereby assess the number of clusters within the data, we performed Gaussian mixture modelling for *k* = 1, 2, 3 and 4 components. For each of the four models, we computed the Bayes information criterion (BIC)^65^, which is an approximation of the integrated likelihood of the converged model. The BIC is defined as twice the negative log of the maximized model likelihood penalized by the addition of the number of unknown parameters multiplied by the log of the number of datapoints. This penalty term is to prevent overfitting of the data by continually increasing model complexity by adding an ever-greater number of parameters. A lower BIC indicates a better-fitting model. We found that the BIC value was lowest (685) for *k* = 1 and progressively increased as more components were added, indicating that the data are best fitted by a single Gaussian distribution and are not statistically separable into clusters. This result was insensitive to whether the covariance matrices were full or diagonal, and we found that convergence was achieved within 50 iterations for each model.

### Palaeogeographic reconstruction

The plate tectonic reconstruction of the pre-break-up positions of the East Antarctic and Australian plates at 157 Ma was performed using GPlates software^58^ and a recent plate tectonic model^66^. The East Antarctic continental shelf area was manually cropped to avoid overlap with adjacent continents and aid visualization.

## Online content

Any methods, additional references, Nature Portfolio reporting summaries, source data, extended data, supplementary information, acknowledgements, peer review information; details of author contributions and competing interests; and statements of data and code availability are available at 10.1038/s41561-025-01734-z.

## Supplementary information

Supplementary Information Supplementary Figs. 1–5, Text 1–5 and References.

## Figures and Tables

**Extended Data Table 1:**
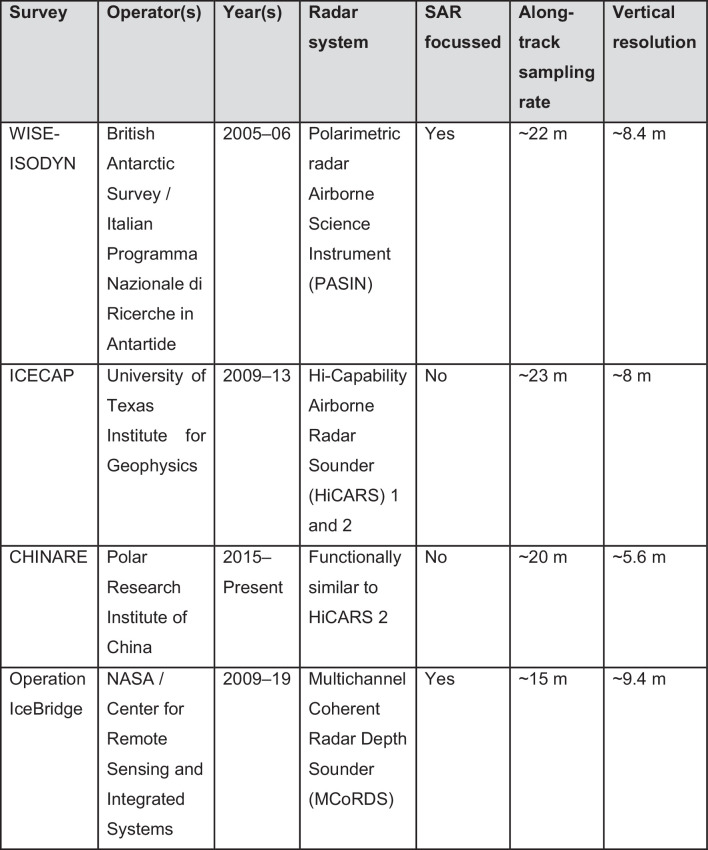
Airborne radar survey details

Characteristics of the airborne radar surveys and platforms from which bed elevation data used in this study were derived. Years show the total duration of the surveys; in some cases, the data used in this study were from a subset of these years, depending on whether lines were flown over the region of interest.

**Extended Data Table 2:**
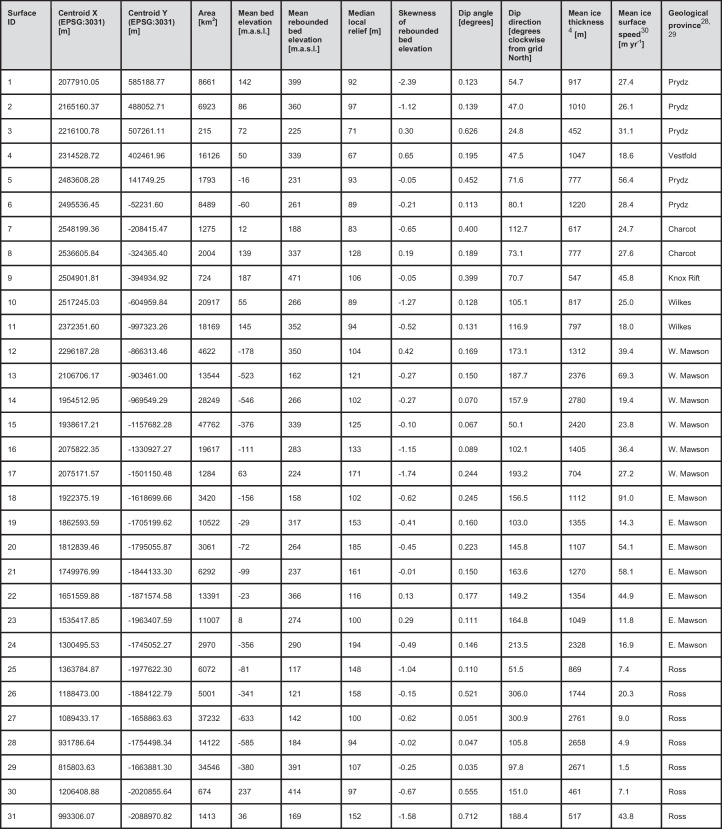
Summary statistics for the 31 flat surfaces mapped around the East Antarctic margin

Spatial coordinates, surface areas and directional data are computed in the EPSG:3031 Antarctic Polar Stereographic projection. Elevations are in metres relative to global mean sea level (m.a.s.l.). Surface IDs correspond to Extended Data Fig. 3; geological provinces are shown in Extended Data Fig. 6.

## Data Availability

The radio-echo sounding bed pick data used in this study are available via WISE-ISODYN^23^, ICECAP^67^^,68^, CHINARE^69^ and OIB^70^. Other geospatial datasets used in this study are available via BedMachine Antarctica^71^, MEaSUREs Antarctic ice velocity^72^, isostatic response to ice-sheet unloading^73^, Copernicus Global 90 m digital elevation model^62^, basal thermal state derived from RES^74^ and ISSM basal thermal state output^75^. The Copernicus/ESA Sentinel-2b images shown in Extended Data Fig. 4 were acquired free of charge from the Copernicus Open Access Hub (https://scihub.copernicus.eu/). The datasets generated as part of this study, including the database of East Antarctic flat surfaces, are available via Zenodo at 10.5281/ZENODO.11367659 (ref. ^76^).
