# Supplementary material for: Extensive fluvial surfaces at the East Antarctic margin have modulated ice-sheet evolution
Source: Nat Geosci. 2025 Jul 11;18(8):724–31. doi: 10.1038/s41561-025-01734-z (PMC12331516; doi:10.1038/s41561-025-01734-z)
Supplement: Supplementary file 1 — Supplementary Figs. 1–5, Text 1–5 and References. [file 41561_2025_1734_MOESM1_ESM.pdf]

nature geoscience

Supplementary information https://doi.org/10.1038/s41561-025-01734-z

# Extensive fluvial surfaces at the East Antarctic margin have modulated ice-sheet evolution

In the format provided by the authors and unedited

## Supplementary Text 1: Isostatic response to Antarctic Ice Sheet unloading

We adjusted bed elevations of the flat surfaces for the isostatic response to the complete unloading of the Antarctic Ice Sheet (Fig. S1a). To do so, we used a recent calculation of the isostatic response to complete deglaciation[1], which was computed using a flexed elastic plate model[2], BedMachine v3 ice thickness[3] (Fig. S1b), and a laterally variable effective elastic thickness of the lithosphere[4]. The correction (Fig. S1c) also accounts for the equilibration of the ongoing response to Antarctic ice mass change since the Last Glacial Maximum and feedbacks associated with loading of areas below sea level by water.

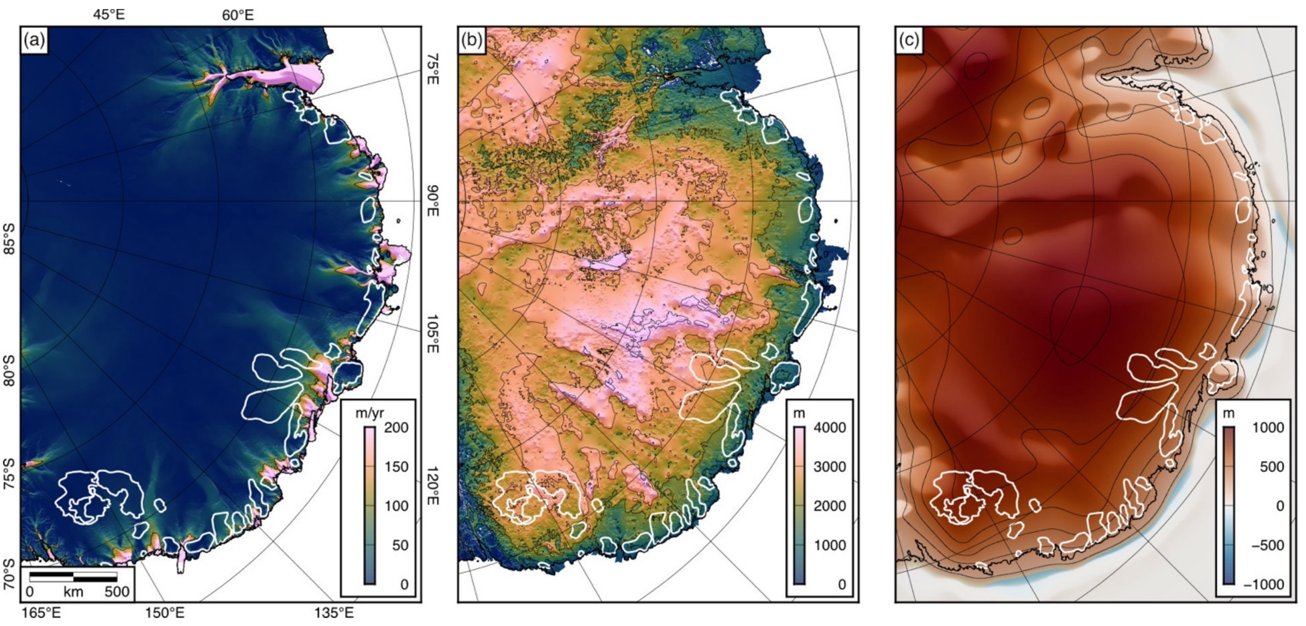


**Fig. S1. Present-day East Antarctic Ice Sheet configuration in the study area.** (a) Ice surface velocity[5], (b) Ice thickness[3], (c) Isostatic response to unloading of the modern ice sheet[1]. White polygons mark the extent of the flat surfaces mapped in this study.

## Supplementary Text 2: Cenozoic sea-level change

Under ice-free conditions (i.e., when elevations are adjusted for the removal of the present-day ice load), the modal flat surface elevations are 200–450 metres above modern-day sea level (Fig. 2 in the main manuscript). However, when the elevations are extrapolated to the modern grounding line they are consistently situated ~150 metres above sea level (Fig. S2). The coastward edge of the flat surfaces is unlikely to be a remnant of a once-larger surface that originally extended out onto the modern continental shelf due to the continuity of the position of the +150 m contour around margin (Fig. S2a). This inference is also supported by the observation that the flat surfaces appear to terminate in a sharp 'cliff' at their seaward edge (rather than continuing offshore; Fig. S5b), although

the near-coastal bathymetry of the East Antarctic margin is poorly known, making this difficult to ascertain with certainty. If this is the case, it implies that the coastline was in approximately the same position as the present-day grounding line prior to glaciation, and the edge of the flat surfaces at +150 m likely records the base level to which the surfaces were eroded. We interpret the surfaces as having originally formed via fluvial planation prior to ca. 34 Ma, and that this palaeo-base level has been 'frozen in' because of the growth of the East Antarctic Ice Sheet and preservation of the flat surfaces (aside from minor surficial modification via areal scouring).

In an ice-free world, global mean geocentric sea level (GMGSL) would have been higher than today due to a combination of barystatic (BSL), thermosteric (TSL), and ocean basin volume (OBVSL) change[6].

$$GMGSL = BSL + TSL + OBVSL \quad (1)$$

Reconstructed sea-level curves for the Cenozoic Era (ca. 66 Ma to present; Fig. S2b) show that GMGSL was 150 ± 50 metres above present-day sea level during the Palaeocene and early Eocene (ca. 66–48 Ma)[6]. The contributions to the higher GMGSL at this time were:

- ~50–120 m from ocean basin volume change[7] (Fig. S2c).
- ~10 m from thermosteric change due to average deep-ocean temperatures being up to 14°C warmer than today[6,8] (Fig. S2d).
- ~50–70 m from barystatic change due to the absence of the Antarctic and Greenland Ice Sheets[3,9] (Fig. S2e).

Gravitational and rotational changes associated with the removal of the Antarctic Ice Sheet would cause an additional relative sea level rise of ~20 metres around Antarctica[1].

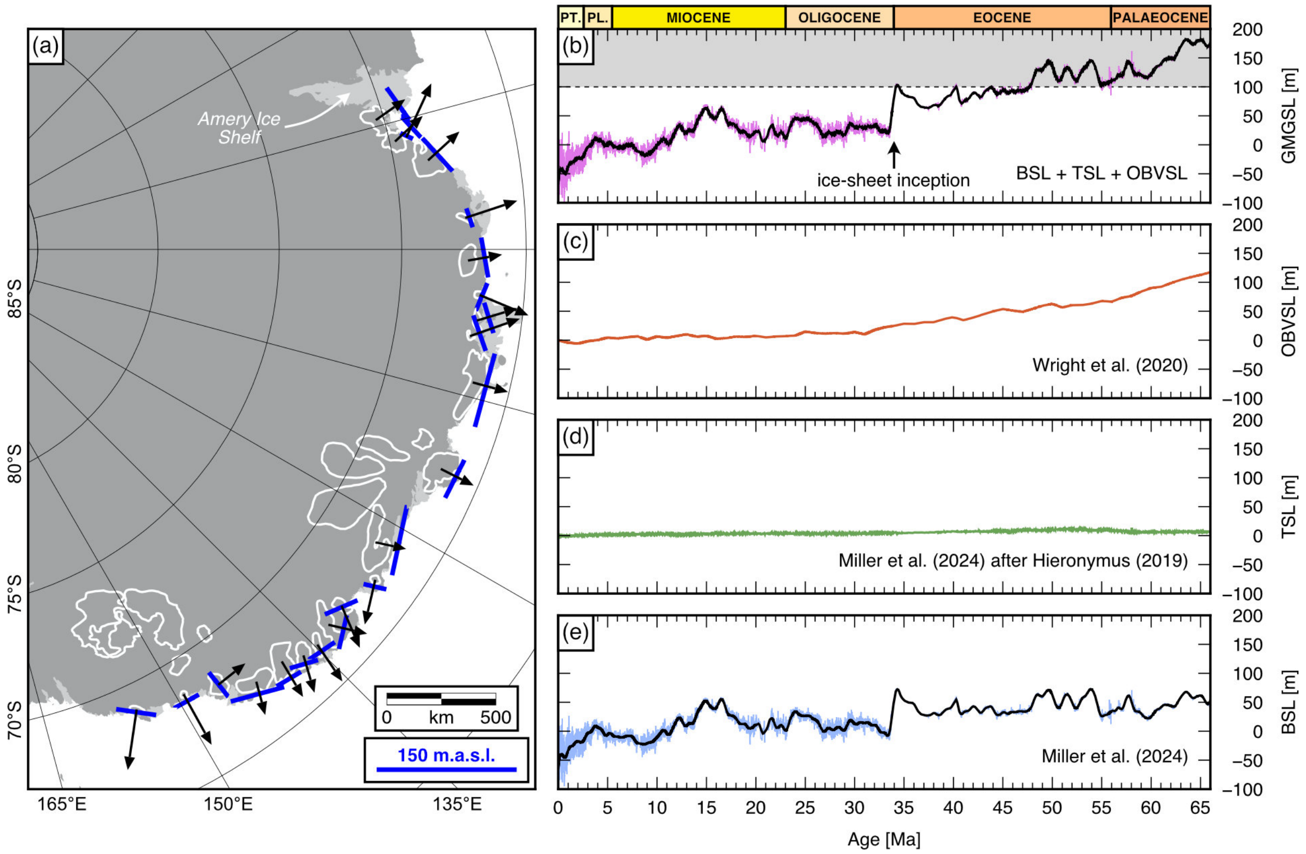


**Fig. S2. Base level recorded by the flat surfaces.** (a) The locations where the near-coastal surfaces reach 150 m above present-day sea level (blue lines). These positions were determined using linear extrapolation of the rebounded bed elevation data within the flat surface polygons (white), assuming the flat surfaces have a constant dip. Vectors indicate the dip direction of the surfaces, with lengths proportional to the dip angle. (b) Global mean geocentric sea level (GMGSL) curve for the Cenozoic[6]. This curve is determined by summing the contributions of the three panels below. (c) Ocean basin volume sea level (OBVSL) change. (d) Thermosteric sea level (TSL) change. (e) Barystatic sea level (BSL) change. In panel b, the grey shaded area bounded by the dashed line marks the GMGSL range of 100–200 m, which is broadly consistent with the implied base level for the flat surfaces (~150 m). The purple and blue lines in panels b and e show the time series of GMGSL and BSL, respectively. The black lines show the trend computed using a LOESS algorithm within an 800 kyr window[6]. Pt. = Pleistocene; Pl. = Pliocene.

The above calculations assume that the flat surfaces have not been vertically displaced after their formation (other than by the isostatic response to ice sheet loading). Other processes may have been capable of vertically displacing the flat surfaces after their formation, including: (i) the isostatic response to glacial trough incision and offshore sediment deposition, (ii) thermal subsidence during the post-rift phase following Australia-Antarctica break-up, and (iii) changes in dynamic topography driven by viscous tractions imparted on the base of the lithosphere arising from convection in the Earth's mantle. Although it is not possible to constrain the exact effects of these processes, we performed first-order calculations to quantify their approximate impact on flat surface elevations.

**Supplementary Text 3: Isostatic response to erosional unloading and sediment loading**

We used a 1D elastic plate model to compute the flexure, $w$, induced by the erosion of the glacial troughs that bound the flat surfaces and the deposition of glacial sediment on the adjacent continental shelf. The 1D flexure equation is expressed as:

$$D\frac{d^4w}{dx^4} + (\rho_{mantle} - \rho_{infill})gw(x) = (\rho_{load} - \rho_{displace})gh(x) \qquad (2)$$

where $h$ is the thickness of the load as a function of distance ($x$), and:

$$D = \frac{E\,Te^3}{12(1-\upsilon^2)} \qquad (3)$$

The parameter Te represents the effective elastic thicknesses (a proxy for flexural rigidity, $D$) of the East Antarctic lithosphere. For simplicity, we assumed a spatially-uniform Te, allowing Eq. (2) to be solved analytically using a fast Fourier transform of the erosional unload and convolution with a 1D flexural isostatic response function[2]. We assumed a Young's modulus of 100 GPa, a Poisson ratio of 0.25, a gravitational acceleration of 9.81 m $s^{-2}$ and densities of 2300, 2700 and 3330 kg $m^{-3}$ for the sediment, eroded material, and mantle, respectively. Note for erosional unloading, the material infilling and displaced by the flexure is air (density 0 kg $m^{-3}$), whereas for sediment loading this material is water (density 1030 kg $m^{-3}$). We used a Te value of 30 km, based on estimates for this section of the East Antarctic margin[4,10], and also performed the calculation using values of 10 km and 50 km to test the sensitivity of the flexural response to lithospheric rigidity.

For the erosional unloading calculation, we used a section of the transect of the East Antarctic margin. For simplicity, the thickness of glacial incision was assumed to be the difference between the observed topography (adjusted for ice-sheet unloading) and sea level (Fig. S3a). We found that although the flexural uplift induced by glacial incision may approach 1000 m over the troughs, minimal uplift (<50 m) is experienced across much of the flat surfaces (Fig. S3b). The exception is the predicted short-wavelength (~50 km) upwarping of the edges of the surfaces, and there is suggestion of this being present in parts of the profile (Fig. S3c) Overall, however, the effect of erosional unloading on the elevations of the flat surfaces is minimal.

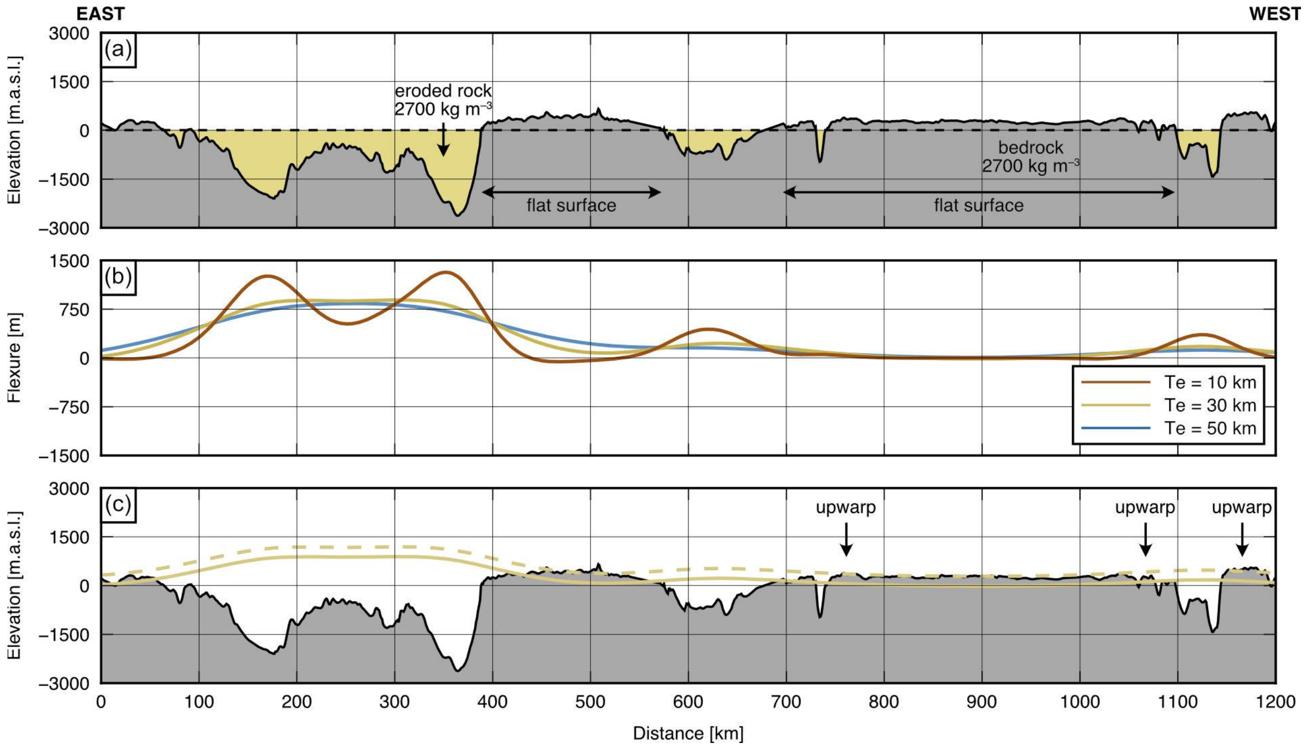


**Fig. S3. Influence of the isostatic response to erosional unloading on flat surface elevations.** (a) Bed elevation, adjusted for ice-sheet loading, from a section of the East Antarctic margin transect (1875–3075 profile-km of Fig. 3b in the main manuscript) sampled from BedMachine v.3[3]. The thickness of glacial incision is assumed to be the difference between sea level (dashed line) and the bed (shaded in yellow). (b) Flexural response to the unloading of the glacially eroded rock (shown in panel a) for three spatially-uniform Te values. (c) Comparison of the bed topography with the flexure pattern (for Te = 30 km). The dashed line shows the flexure vertically shifted by +300 metres to allow comparison with the shape of the flat surfaces. Note that the uplift over the flat surfaces is minimal other than short-wavelength upwarping near their trough-bounded edges.

For the sediment loading calculation, the paucity of seismic reflection data between the continental shelf edge and the coast meant it was not possible to use a true sediment thickness profile. Instead, we constructed a simple 1D model of sediment thickness based on available constraints from the Adélie–Wilkes Land margin. Specifically, compilations of seismic reflection data indicate that the maximum thicknesses of glacially-derived (post-34 Ma) sediment are ~3 km and these occur ~300 km from the edge of the flat surfaces[11] (Fig. S4a). Additionally, the small number of seismic profiles acquired over the continental shelf in George V Land and Adélie Land indicate that the first basement 'outcrops' (i.e., sediment thickness = 0 km) are typically ~20 km from the coast, and post-34 Ma sediment thicknesses increase to ~1 km at the shelf edge, ~150 km from the coast[12] (Fig. S4a). We found that, irrespective of the Te value, the subsidence induced by sediment loading does not propagate sufficiently far inland to have an appreciable impact (<50 m) on flat surface elevations (Fig. S4b) and cannot account for the observed amplitude and wavelength of their coastward dips (Fig. S4c).

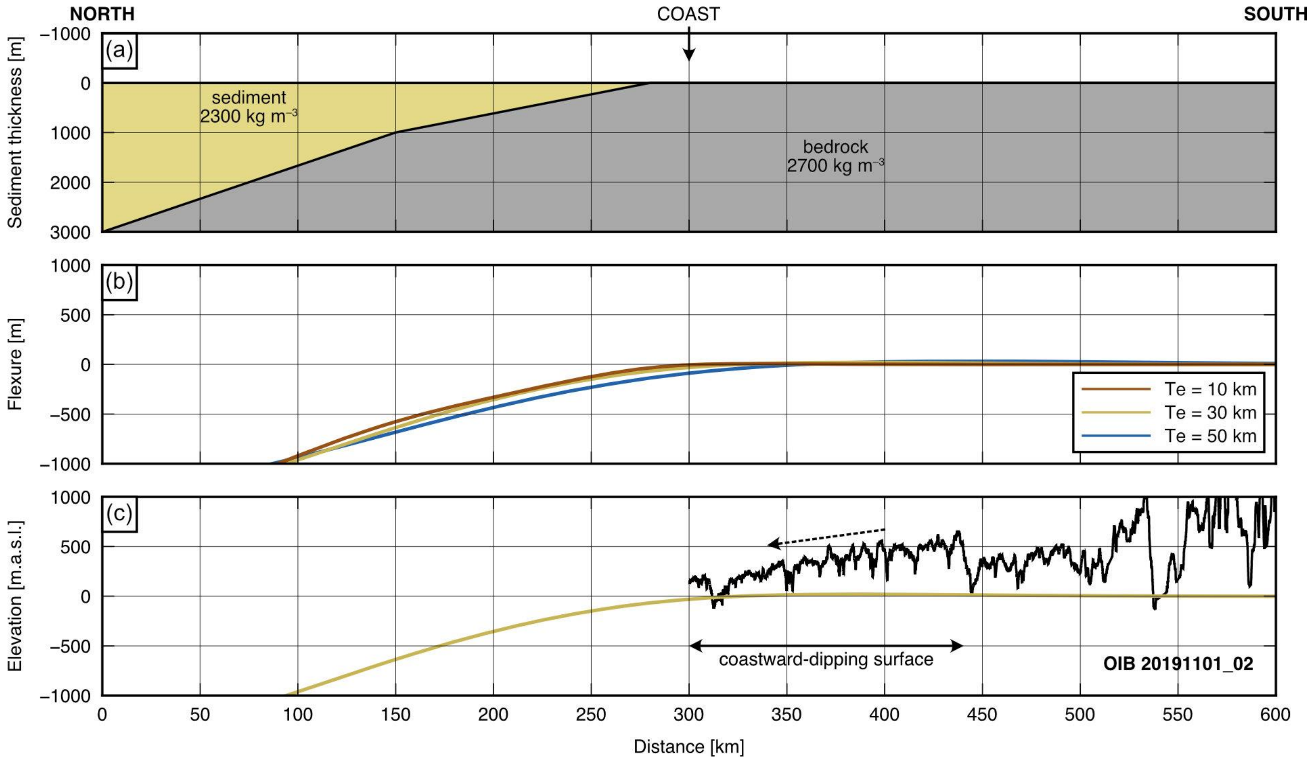


**Fig. S4. Influence of the isostatic response to sediment loading on flat surface elevations.** (a) Idealised model of offshore sediment thickness for a profile oriented perpendicular to the coast. The position of the coast is labelled. See the text for description of how the geometry of the glacial sediment (yellow region) was established. (b) Flexural response to the loading of the glacial sediment (shown in panel a) for three spatially-uniform Te values. (c) Comparison of the RES-derived bed topography (Operation IceBridge flight 20191101_02; adjusted for ice loading) of a coastward-dipping surface in Adélie Land (see Extended Data Fig. 2) with the flexure pattern (for Te = 30 km). Note that the impact of the subsidence on the flat surface is minimal and cannot account for the amplitude or wavelength of its coastward dip, irrespective of the Te value.

## Supplementary Text 4: Post-rift thermal subsidence

To estimate the impact of post-rift thermal subsidence on flat surface elevations, we used a simple 1D cooling model for crustal extension[13]. The post-rift subsidence, as a function of time (S(t)), is given by:

$$S(t) = E_0\, r\,(1 - exp(-t/\tau)) \tag{4}$$

where

$$E_0 = \frac{4y_L\rho_m\alpha_V(T_m - T_0)}{\pi^2(\rho_m - \rho_i)} \tag{5}$$

and

$$r = \frac{\beta}{\pi} sin(\frac{\pi}{\beta}) \tag{6}$$

and

$$\tau = \frac{{y_L}^2}{\pi^2 \kappa} \quad (7)$$

We assumed typical values for the fixed parameters in these equations, including a lithosphere thickness ($y_L$) of 125 km, mantle density ($\rho_m$) of 3330 kg $m^{-3}$, volumetric coefficient of thermal expansion ($\alpha_v$) of 3.28 x $10^{-5}$ $K^{-1}$, mantle temperature relative to the surface ($T_m$–$T_0$) of 1333 K, thermal diffusivity ($\kappa$) of $10^{-6}$ $m^2$ $s^{-1}$. The material infilling the subsidence was assumed to be air ($\rho_i$ = 0 kg $m^{-3}$), since the flat surfaces would have been subaerial. The stretching factor ($\beta$) indicates the amount of extension. Although this is poorly constrained for the East Antarctic margin, estimates for Wilkes Land suggest that $\beta$ decreases from a value of ~5 at the continent-ocean boundary to <1.1 at the coast, ~350 km away[10] (Fig. S5a). Since the flat surfaces are inboard of the coast (Fig. S5b), we assume that the stretching factor is unlikely to have exceeded 1.1 in their vicinity. We assumed that the post-rift phase began no later than 80 Ma, based on the age of the oldest seafloor in the Australian-Antarctic sector[14]; we computed the cumulative thermal subsidence from 80 Ma to the present-day.

Based on these assumptions, the predicted post-80 Ma thermal subsidence experienced by the flat surfaces is <170 m, with only ~70 m of that subsidence occurring after ca. 50 Ma (Fig. S5c). The maximum subsidence is likely to have occurred at the coastward edges of the flat surfaces, gradually decreasing moving further inland. Although there is no geological evidence to confirm the magnitude of post-rift subsidence of the East Antarctic margin, these predictions are in good agreement with backstripping analysis from the conjugate southern Australian margin, which indicates that Cenozoic thermal subsidence was minor or even negligible at many sites[15].

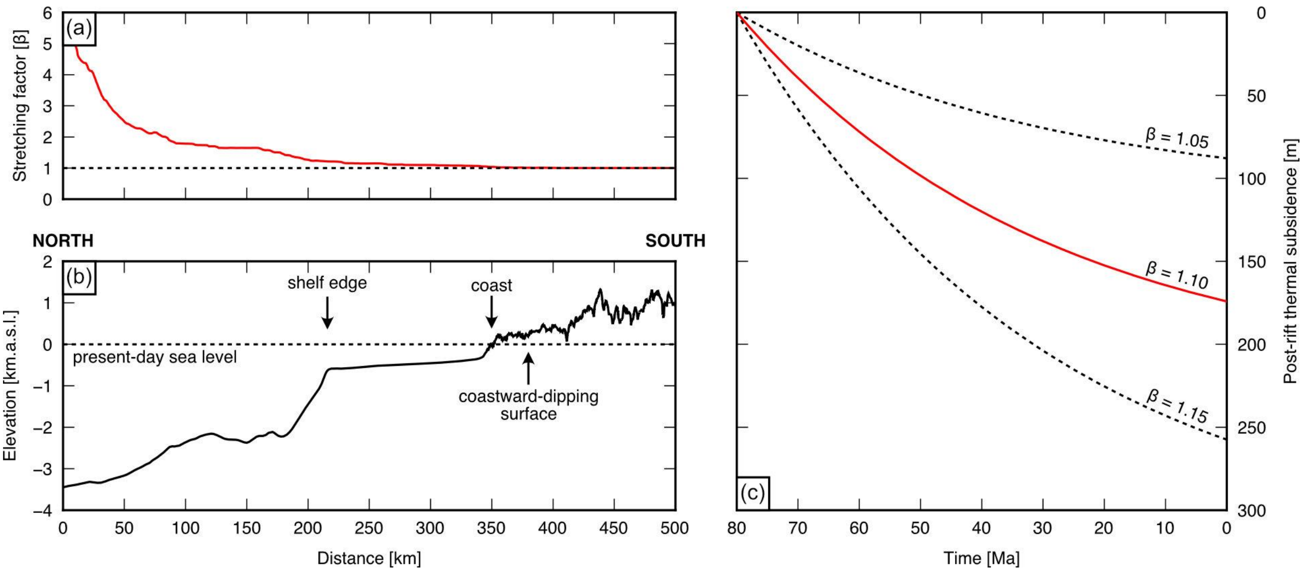


**Fig. S5. Influence of post-rift thermal subsidence on flat surface elevations.** (a) Variation of crustal stretching factor (β) with distance from the continent-ocean boundary[10]. Dashed line marks β = 1 (i.e., zero extension). (b) Bathymetric and topographic profile. Elevations have been adjusted for modern ice sheet loading. Bathymetric data were derived from the International Bathymetric Chart of the Southern Ocean[16]; bed elevation data were derived from ICECAP RES survey line IR2HI2_2011018_ASB_JKB2d_GL0375a. The position of the coast and continental shelf edge are labelled. (c) Post-rift (ca. 80–0 Ma) thermal subsidence for stretching factors of 1.10 (red) and 1.05 and 1.15 (dashed black) computed using a 1D model of crustal extension[13].

## Supplementary Text 5: Cenozoic vertical land motion and pattern of flat-surface elevations

Our calculations demonstrate that none of the processes considered above has had a significant impact (>50 m) on the elevations of the flat surfaces since ice sheet inception at ca. 34 Ma. Moreover, these processes generate displacements with opposite signs, meaning minor uplift caused by erosional unloading will have been at least partially offset by minor subsidence caused by sediment loading and post-rift cooling (or vice versa). Importantly, the patterns of displacement caused by these processes cannot account for the consistent long-wavelength gentle coastward dips of the flat surfaces, meaning this is an original characteristic arising from their formation. Furthermore, the consistency in the grading of the flat surfaces to 150 ± 50 metres above sea level (which approximates Palaeocene–early Eocene sea level) around the entire margin indicates that the surfaces have not experienced substantial *differential* uplift or subsidence since their formation.

The apparent base level recorded by the flat surfaces is therefore broadly consistent with the estimated sea-level for the Palaeocene to early Eocene (ca. 66–48 Ma), which falls within our proposed age range for flat surfaces based on plate tectonic, stratigraphic, and geomorphological evidence (ca. 80–34 Ma). Sea-level fall from the late Eocene onwards was driven primarily by the

barystatic effect of the growth of the Antarctic Ice Sheet (Fig. S2b,e). Formation of the ice sheet would have also prevented new flat coastal surfaces from being planed to the new, lower base level by fluvial systems after ca. 34 Ma.

**References**


1. Paxman, G. J. G., Austermann, J. & Hollyday, A. Total isostatic response to the complete unloading of the Greenland and Antarctic Ice Sheets. *Sci. Rep.* **12**, 11399 (2022).
2. Watts, A. B. *Isostasy and Flexure of the Lithosphere*. (Cambridge University Press, Cambridge, 2001).
3. Morlighem, M. *et al.* Deep glacial troughs and stabilizing ridges unveiled beneath the margins of the Antarctic ice sheet. *Nat. Geosci.* **13**, 132–137 (2020).
4. Swain, C. J. & Kirby, J. F. Effective Elastic Thickness Map Reveals Subglacial Structure of East Antarctica. *Geophys. Res. Lett.* **48**, e2020GL091576 (2021).
5. Mouginot, J., Rignot, E. & Scheuchl, B. Continent-Wide, Interferometric SAR Phase, Mapping of Antarctic Ice Velocity. *Geophys. Res. Lett.* **46**, 9710–9718 (2019).
6. Miller, K. G. *et al.* Global Mean and Relative Sea-Level Changes Over the Past 66 Myr: Implications for Early Eocene Ice Sheets. *Earth Sci. Syst. Soc.* **3**, 10091 (2024).
7. Wright, N. M., Seton, M., Williams, S. E., Whittaker, J. M. & Müller, R. D. Sea-level fluctuations driven by changes in global ocean basin volume following supercontinent break-up. *Earth-Sci. Rev.* **208**, 103293 (2020).
8. Hieronymus, M. An update on the thermosteric sea level rise commitment to global warming. *Environ. Res. Lett.* **14**, 054018 (2019).
9. Morlighem, M. *et al.* BedMachine v3: Complete Bed Topography and Ocean Bathymetry Mapping of Greenland From Multibeam Echo Sounding Combined With Mass Conservation. *Geophys. Res. Lett.* **44**, (2017).
10. Close, D. I., Watts, A. B. & Stagg, H. M. J. A marine geophysical study of the Wilkes Land rifted continental margin, Antarctica. *Geophys. J. Int.* **177**, 430–450 (2009).

11. Hochmuth, K. *et al.* The Evolving Paleobathymetry of the Circum-Antarctic Southern Ocean Since 34 Ma: A Key to Understanding Past Cryosphere-Ocean Developments. *Geochem. Geophys. Geosystems* **21**, e2020GC009122 (2020).
12. De Santis, L., Brancolini, G., Donda, F. & O'Brien, P. Cenozoic deformation in the George V Land continental margin (East Antarctica). *Mar. Geol.* **269**, 1–17 (2010).
13. McKenzie, D. Some remarks on the development of sedimentary basins. *Earth Planet. Sci. Lett.* **40**, 25–32 (1978).
14. Müller, R. D. *et al.* Ocean Basin Evolution and Global-Scale Plate Reorganization Events Since Pangea Breakup. *Annu. Rev. Earth Planet. Sci.* **44**, 107–138 (2016).
15. Hegarty, K. A., Weissel, J. K. & Mutter, J. C. Subsidence History of Australia's Southern Margin: Constraints on Basin Models. *AAPG Bull.* **72**, (1988).
16. Arndt, J. E. *et al.* The International Bathymetric Chart of the Southern Ocean (IBCSO) Version 1.0—A new bathymetric compilation covering circum-Antarctic waters. *Geophys. Res. Lett.* **40**, 3111–3117 (2013).
